# Methods for measuring stratigraphic attitude and true thickness based on high-precision 3D real-scene models: A case study of the early Silurian Chongqing Lagerstätte Section, China

**DOI:** 10.1371/journal.pone.0358199

**Published:** 2026-09-18

**Authors:** Ruigang Zhang, Zhiwei Cui, Yang Chen, Jinhua Luan, Xiong Zhang, Dan Zhou, Deju Zhang, Haitong Zhao, Zhengqin Gan, Xinzhe Liu

**Affiliations:** Chongqing Key Laboratory of Resource and Environmental Effects of Major Geological Events (Chongqing Institute of Geology and Mineral Resources), Chongqing, China; Sichuan University of Science and Engineering, CHINA

## Abstract

Accurate measurement of stratigraphic attitude and true thickness is critical to geological exploration, mineral resource assessment, and geological disaster prevention. Traditional measurement methods are limited by accuracy and field conditions. With the development of high-precision 3d real-scene modeling technologies, a new solution for these measurements has emerged. This study aims to use high-precision 3D real-scene models to measure stratigraphic attitude and true thickness, and to validate the reliability and practicality. First, high-precision 3D real-scene models were constructed using UAV oblique photography data. Then, we can calculate the normal vector based on three non-collinear points on a plane. We can calculate the attitude and equation of this plane based on the normal vector. The true thickness of strata between two planes was calculated using the formula for the distance from any point on another plane in space to the plane equation. Finally, the methods used to measure the fish fossil-bearing section in Xiushan, Chongqing, China. So, we used this new method to measure 80 stratigraphic joint attitudes and the true thickness of 7 strata. Then, we compared these measurements with 20 stratigraphic joint attitudes and the true thickness of the same 7 strata that we got using traditional methods. The results show that stratigraphic attitude and true thickness obtained using high-precision 3D real-scene models are reliable, and this method can overcome the limitations of traditional measurement methods. This study provides a new method for measuring stratigraphic attitude and true thickness, with good application potential and practical value.

## 1 Introduction

It’s important for geological survey and study to accurately measure stratigraphic attitude and true thickness. Such as when conducting regional geological surveys, exploring for mineral resources and evaluating geological disasters [1–5]. However, using compasses and tape measures to measure stratigraphic attitude and true thickness is inefficient, provides poor accuracy, and is greatly limited by field conditions [6,7]. Therefore, it is necessary to develop more efficient, accurate, and convenient measurement methods. With the advancements in UAV oblique photography and high-precision 3D real-scene modeling technologies, these techniques have been widely applied in geological mapping, geological disaster evaluation, soil quality surveys, digital section construction and online field trip [8–15].While UAV-based 3D modeling has been applied in geological studies, previous attempts to measure stratigraphic attitude and true thickness often still relied on traditional calculation formulas. This approach can introduce systematic errors, involve complex calculation processes, and ultimately hinder practical application in field geological work. This study addresses these shortcomings by integrating simple spatial geometric algorithms with high-precision 3D real-scene models for accurate and efficient stratigraphic attitude and true thickness calculations. The discovery of the Chongqing Lagerstätte reveals a previously unseen diversification of jawless and jawed vertebrates in the early Silurian [16]. The methodology will be applied to measure and analyze the Silurian strata in Xiushan, Chongqing, China, to validate its feasibility and effectiveness, proposing a more precise and efficient method for measuring strata attitude and true thickness. The method allows for the remote measurement of strata attitude and thickness without physical contact, which is particularly important for inaccessible areas in field geological work. Moreover, this approach is expected to significantly reduce the time and resources needed for field measurements, while providing more accurate and reliable data than traditional methods.

## 2 Geological background

**Fig 1:**
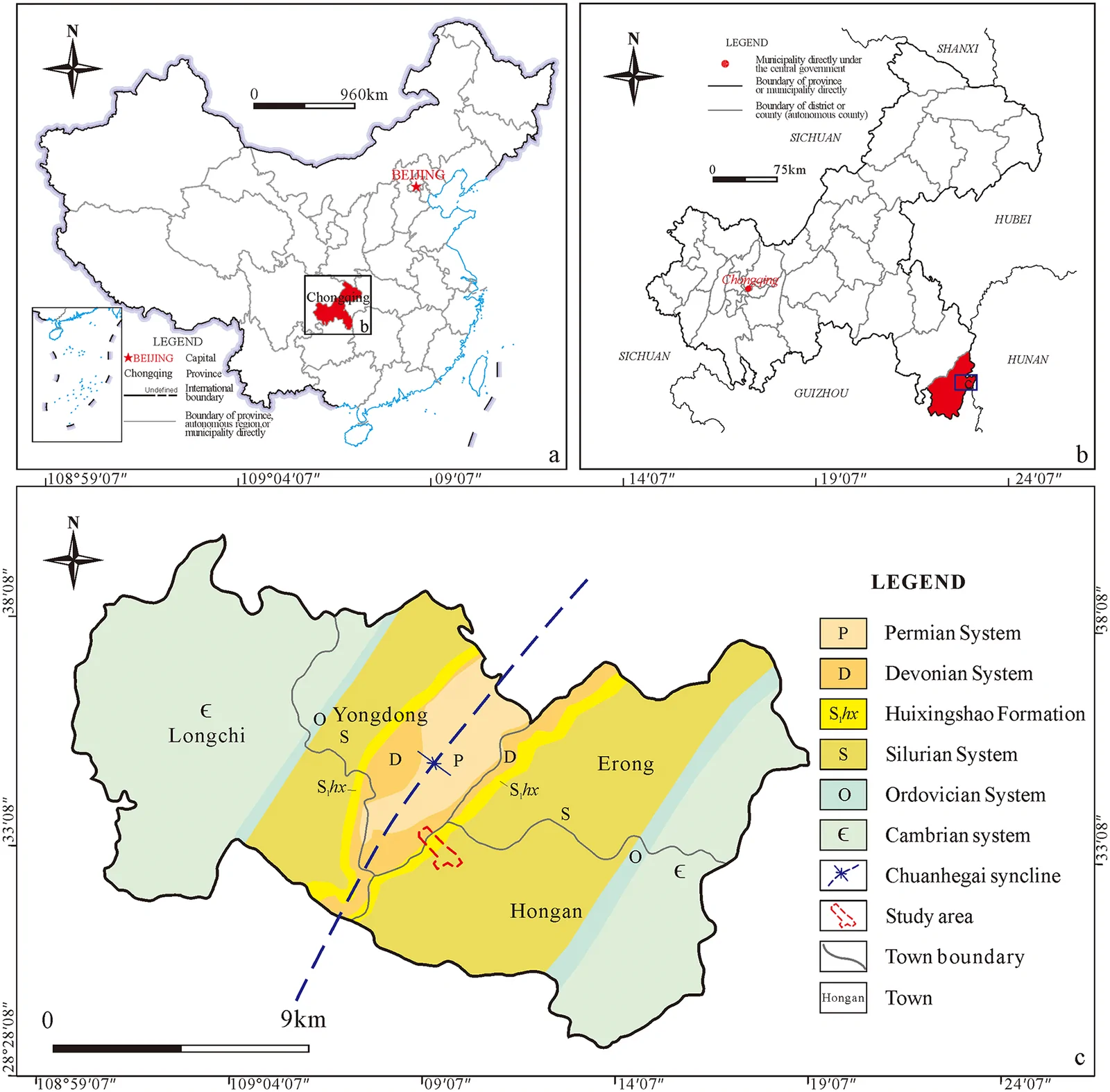
Simplified Geological map of the study area (Chen et al., 2022).

The study area is located at Tianlu in Hongan Town, Xiushan County, Chongqing, China. The geotectonic position is located at the east wing of Chuanhegai syncline in Youxiu fold belt. The strata belong to the Huixingshao Formation of the Llandovery Series, Silurian (Fig 1) [17]. The lower part of this formation develops thin to medium bedded red sandstone, muddy sandstone, and sandy mudstone, collectively known as the upper red beds. The middle to upper parts develops medium to thick bedded grey-yellow sandstone and muddy sandstone with joint development due to local structural effects. As the area yields early Silurian fish fossils, the research team has conducted multiple geological surveys and section measurements here [17,18]. To acquire detailed geological characteristics such as stratigraphic features, structural features, and fossil distributions, extensive geological measurements are necessary, including the measurement of attitude, joint attitude, and true thickness of strata. Due to the steep nature of the geological outcrops and the high positions of key geological information within the strata, comprehensive geological measurements are challenging. Therefore, UAV oblique photography is used to aerially photograph the target strata in the study area, followed by high-precision 3D real-scene models, aiming for efficient, rapid, comprehensive, and accurate acquisition of key geological information.

## 3 Data collection

### 3.1 UAV (Unmanned Aerial Vehicle) oblique photography

Data collection was conducted using the DJI M3 series professional UAV in this study. Two methods of UAV oblique photography were employed: automated flight and manual flight [14,19] For automated flight, the planned data collection area was uploaded into the UAV’s flight control system, with an aerial photography altitude set to 60 meters and a flight path overlap of 75 percent. The UAV flight control system automatically collected oblique photographs from five directions based on the planned route and default flight speed. The study area covered 1.2 km^2^ (Fig 1), and the UAV flew for 1 hour and 20 minutes, collecting a total of 2,533 photographs. Manual flight collection was conducted over key geological outcrop areas. During manual flight collection, the UAV was flown as close as possible to the geological outcrops, collecting a total of 1,200 photographs.

### 3.2 3D real-scene model

**Fig 2:**
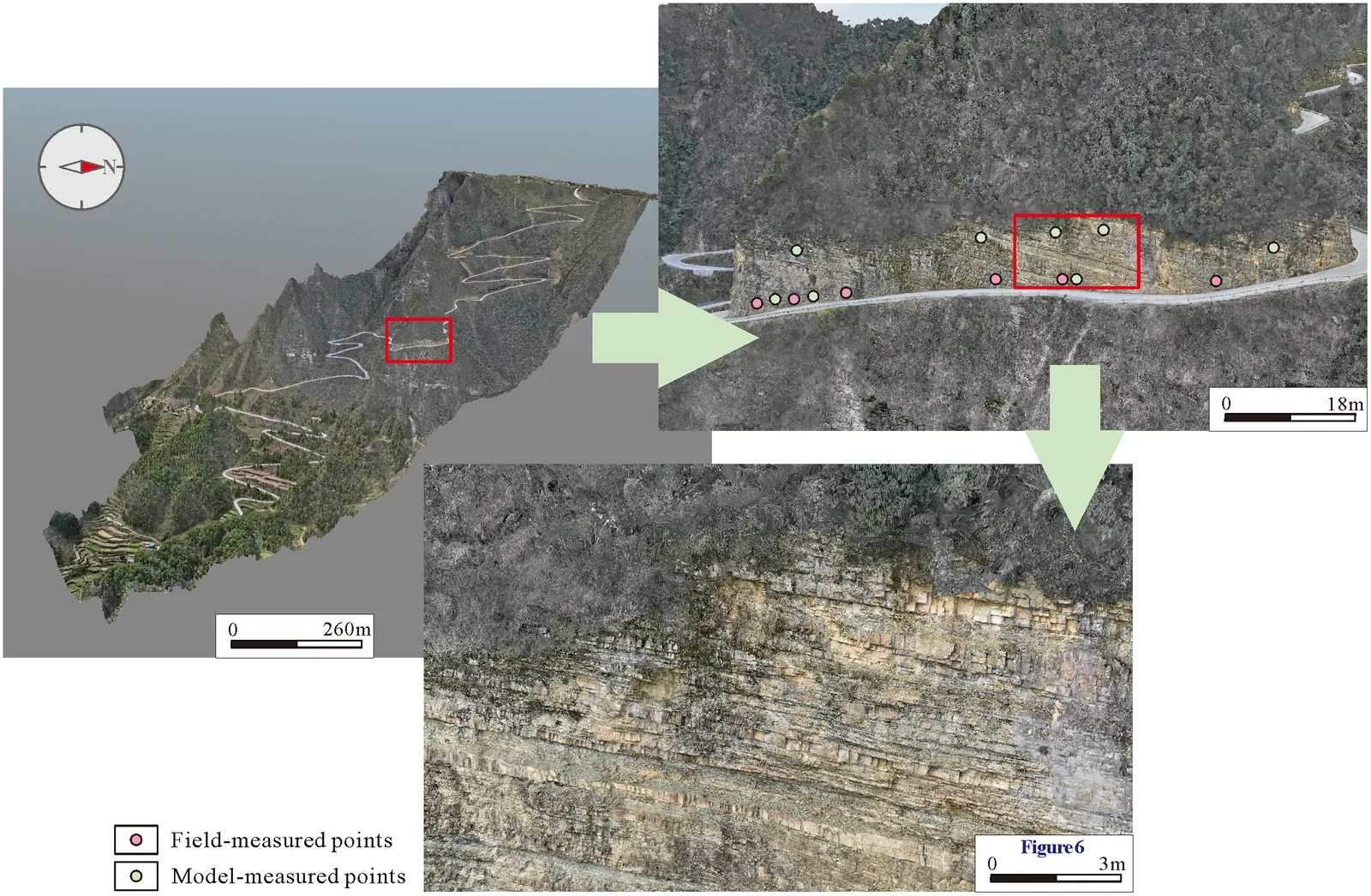
The high-precision 3D real-scene model of the study area.

The 3d real-scene model of the UAV oblique photography data was conducted using DJI Terra software. This process is primarily divided into four stages: the aerial triangulation model stage, the TIN grid model stage, the white body model stage, and the 3D real-scene model stage [14,20,21]. Each of these stages is automated by the software. The constructed model is shown in Fig 2.

## 4 Method

### 4.1 Attitude measurement

**Fig 3:**
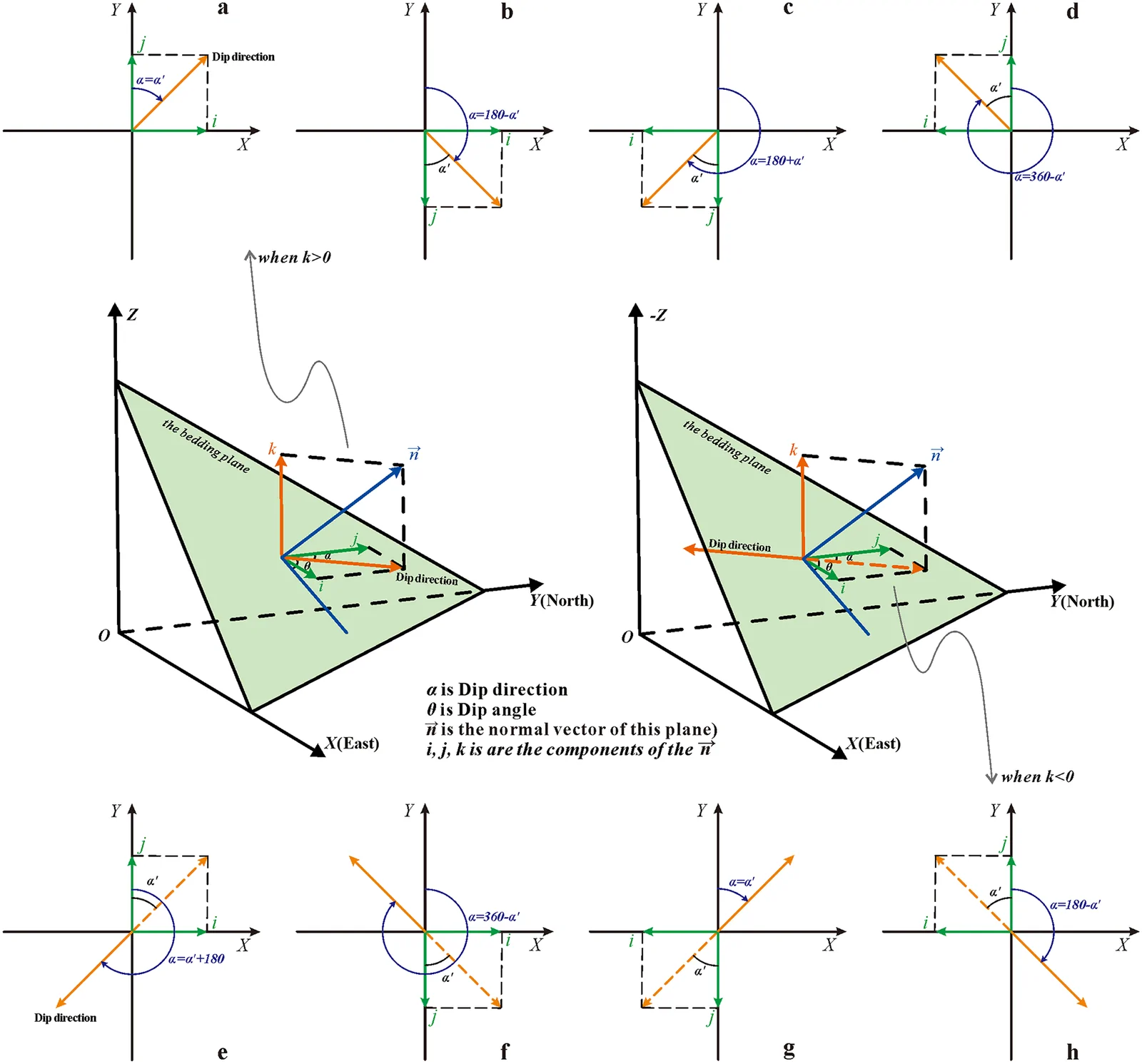
The principle diagram to calculate the attitude of strata.

Stratigraphic attitude includes three elements: trend, dip direction, and dip angle [22,23]. Traditional attitude measurements typically involve the use of mechanical or electronic compasses [24,25]. With the advancement of high-precision positioning equipment, methods have also been developed that use total stations or high-precision GPS devices to measure the coordinates of three non-collinear points on a plane, from which the attitude of the plane is calculated [26–28]. These methods require direct contact between the measuring equipment and the measured plane, which can be nearly impossible in steep terrain. In this study, high-precision 3D real-scene models are used to obtain three non-collinear points on any plane without physical contact, allowing for the calculation of the plane’s attitude. The specific measurement and calculation methods are as follows:

Assume that the coordinates of three non-collinear points on a plane are given as $A\left(x_{\text{1}},y_{\text{1}},z_{\text{1}}\right),$ $B\left(x_{\text{2}},y_{\text{2}},z_{\text{2}}\right)$ and $C\left(x_{\text{3}},y_{\text{3}},z_{\text{3}}\right)$ (Fig 3).

(1) Calculate the normal vector.

Vector $\vec{AB}=B-A=(x_{\text{2}}-x_{\text{1}}{,y}_{\text{2}}-y_{\text{1}},\;z_{\text{2}}-z_{\text{1}})$

Vector $\vec{AC}=C-A=(x_{\text{3}}-x_{\text{1}}{,y}_{\text{3}}-y_{\text{1}},\;z_{\text{3}}-z_{\text{1}})$

Normal vector $\vec{n}=\vec{AB}\times \vec{AC}$=$\left|\begin{array}{ccc} a & b & c\\ x_{\text{2}}-x_{\text{1}} & y_{\text{2}}-y_{\text{1}} & z_{\text{2}}-z_{\text{1}}\\ x_{\text{3}}-x_{\text{1}} & y_{\text{3}}-y_{\text{1}} & z_{\text{3}}-z_{\text{1}} \end{array}\right|$


$$\begin{array}{c} \vec{n}=a\left|\begin{array}{cc} y_{\text{2}}-y_{\text{1}} & z_{\text{2}}-z_{\text{1}}\\ y_{\text{3}}-y_{\text{1}} & z_{\text{3}}-z_{\text{1}} \end{array}\right|-b\left|\begin{array}{cc} x_{\text{2}}-x_{\text{1}} & z_{\text{2}}-z_{\text{1}}\\ x_{\text{3}}-x_{\text{1}} & z_{\text{3}}-z_{\text{1}} \end{array}\right|+c\left|\begin{array}{cc} x_{\text{2}}-x_{\text{1}} & y_{\text{2}}-y_{\text{1}}\\ x_{\text{3}}-x_{\text{1}} & y_{\text{3}}-y_{\text{1}} \end{array}\right| \end{array}$$

Here, *a*, *b*, and *c* denote the unit vectors along the *x*, *y*, and *z* axes, respectively.


$$\begin{array}{c} \vec{n}=\left(\left|\begin{array}{cc} y_{\text{2}}-y_{\text{1}} & z_{\text{2}}-z_{\text{1}}\\ y_{\text{3}}-y_{\text{1}} & z_{\text{3}}-z_{\text{1}} \end{array}\right|,-\left|\begin{array}{cc} x_{\text{2}}-x_{\text{1}} & z_{\text{2}}-z_{\text{1}}\\ x_{\text{3}}-x_{\text{1}} & z_{\text{3}}-z_{\text{1}} \end{array}\right|,\left|\begin{array}{cc} x_{\text{2}}-x_{\text{1}} & y_{\text{2}}-y_{\text{1}}\\ x_{\text{3}}-x_{\text{1}} & y_{\text{3}}-y_{\text{1}} \end{array}\right|\right)=\text{\textasciitilde{}}(i,j,k) \end{array}$$

$$\begin{array}{c} i=\left(y_{\text{2}}-y_{\text{1}}\right)\left(z_{\text{3}}-z_{\text{1}}\right)-(z_{\text{2}}-z_{\text{1}})(y_{\text{3}}-y_{\text{1}}) \end{array}$$

$$\begin{array}{c} j=-(\left(x_{\text{2}}-x_{\text{1}}\right)\left(z_{\text{3}}-z_{\text{1}}\right)-\left(z_{\text{2}}-z_{\text{1}}\right)\left(x_{\text{3}}-x_{\text{1}}\right)) \end{array}$$

$$\begin{array}{c} k=\left(x_{\text{2}}-x_{\text{1}}\right)\left(y_{\text{3}}-y_{\text{1}}\right)-\left(y_{\text{2}}-y_{\text{1}}\right)\left(x_{\text{3}}-x_{\text{1}}\right) \end{array}$$

(2) Calculate the dip angle.

Dip angle (θ) is the angle between the bedding plane and the horizontal plane (Fig. 3), which can be calculated using the following trigonometric function:


$$\begin{array}{c} \theta =\text{90}-arctan\left(\frac{\left|k\right|}{\sqrt{i^{\text{2}}+j^{\text{2}}}}\right)\times \left(\frac{\text{180}}{\pi }\right) \end{array}$$

(3) Calculate the dip direction and trend.

Dip direction (α) is the angle between orientation of the bedding plane and the north direction (Fig 3), which can be calculated using the following trigonometric function:


$$\begin{array}{c} \alpha^{\prime }=\left|arctan\left(\frac{i}{j}\right)\right|\times \left(\frac{\text{180}}{\pi }\right) \end{array}$$

When k>0, i>0, j>0, $\alpha =\alpha^{\prime }$ (Fig 3a).

When k>0, i>0, j<0, $\alpha =\text{180}-\alpha^{\prime }$ (Fig 3b).

When k>0, i<0, j<0, $\alpha ={\text{180}+\alpha }^{\prime }$ (Fig 3c).

When k>0, i<0, j>0, $\alpha ={\text{360}-\alpha }^{\prime }$ (Fig 3d).

When k<0, i>0, j>0, $\alpha =\alpha^{\prime }+\text{1}\text{8}\text{0}$ (Fig 3e).

When k<0, i>0, j<0, $\alpha =\text{360}-\alpha^{\prime }$ (Fig 3f).

When k<0, i<0, j<0, $\alpha =\alpha^{\prime }$ (Fig 3g).

When k<0, i<0, j>0, $\alpha ={\text{180}-\alpha }^{\prime }$ (Fig 3h).

Trend (β) is the angle between the intersection line of the bedding plane with the horizontal plane and the north direction. which can be calculated using the following formula:


$$\begin{array}{c} \beta =\alpha \pm \text{90} \end{array}$$

From the above formula, it is evident that trend has two directions, which differ by 180 degrees.

### 4.2 True thickness measurement

**Fig 4:**
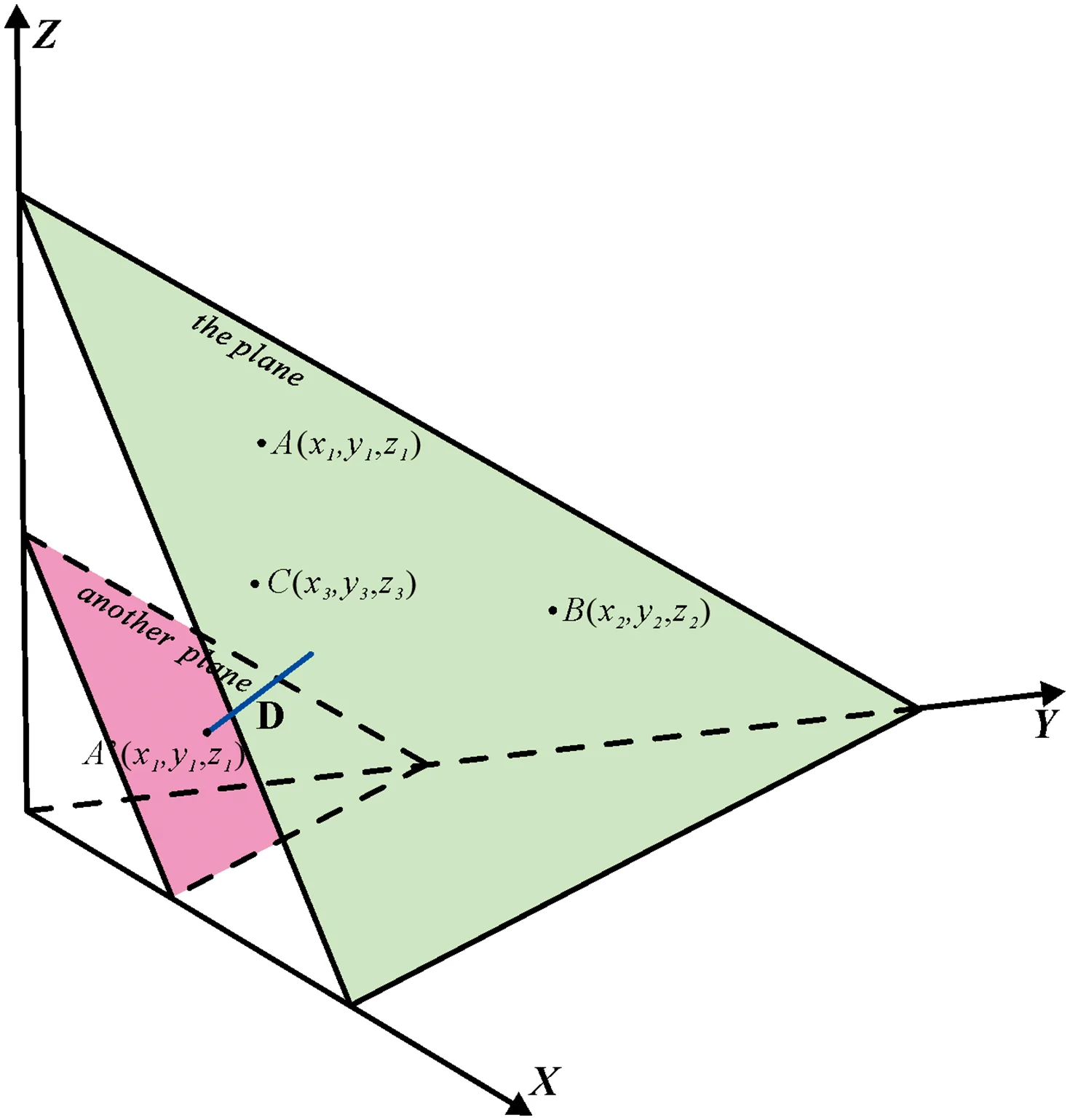
The principle diagram to calculate the true thickness of strata.

Measured stratigraphic section is an important field geological method in geology, widely used in studies of sedimentology, stratigraphy, and the burial of paleontological fossils, among others. The methods for calculating the true thickness of strata have been proposed by numerous scholars from the 1920s to the 1960s [29–33]. In China, the formula is primarily used for calculating the true thickness of strata. However, this formula still has applicability issues after multiple modifications [3,34–37]. With the development of high-precision 3D real-scene modeling technology, it has become easier to obtain the true thickness of strata through simple spatial geometric calculations. The principle is the distance from a point to a plane in spatial geometry (Fig 4), which allows for a more efficient and accurate measurement of the true thickness. The specific measurement and calculation methods are as follows:

Assume that the coordinates of three non-collinear points on a plane are given as $A\left(x_{\text{1}},y_{\text{1}},z_{\text{1}}\right),$ $B\left(x_{\text{2}},y_{\text{2}},z_{\text{2}}\right)$ and $C(x_{\text{3}},y_{\text{3}},z_{\text{3}})$

(1) The method for calculating the normal vector is the same as the method described in chapter 4.1(1).
(2) Calculate the equation of the plane.

$$\begin{array}{c} ix+jy+kz-\left(ix_{\text{1}}+jy_{\text{1}}+kz_{\text{1}}\right)=\text{0} \end{array}$$

(3) Calculate the true thickness D, which is the distance from any point $A\text{'}(x^{\prime },y^{\prime },z^{\prime })$ on another plane to the known plane.

$$\begin{array}{c} D=\frac{\left|ix^{\prime }+jy^{\prime }+kz^{\prime }-\left(ix_{\text{1}}+jy_{\text{1}}+kz_{\text{1}}\right)\right|}{\sqrt{i^{\text{2}}+j^{\text{2}}+k^{\text{2}}}} \end{array}$$

### 4.3 Automated calculation program

Automated calculations can be performed using Excel functions based on the above principles. Simply import the collected spatial point coordinates into the program to obtain the calculation results.

(1) Using Excel’s DEGREES, ATAN, ABS and SQRT functions to calculate the dip angle. The formula is as follows:

=90-DEGREES (ATAN (ABS (k)/SQRT (i^2+j^2)))

(2) Using Excel’s IF, ATAN, DEGREES and ABS functions to calculate the dip direction. The pseudocode is as follows:

IF *k* > 0 THEN

IF *i* > 0 THEN

IF *j* > 0 THEN

result = DEGREES(ABS(ATAN(*i* / *j*)))

ELSE

result = 180 - DEGREES(ABS(ATAN(*i* / *j*)))

END IF

ELSE // *i* <= 0

IF *j* < 0 THEN

result = 180 + DEGREES(ABS(ATAN(*i* / *j*)))

ELSE // *j*>= 0

result = 360 - DEGREES(ABS(ATAN(*i* / *j*)))

END IF

END IF

ELSE // *k* <= 0

IF *i* > 0 THEN

IF *j* > 0 THEN

result = DEGREES(ABS(ATAN(*i* / *j*)))

ELSE // *j* <= 0

result = (180 - DEGREES(ABS(ATAN(*i* / *j*)))) + 180

END IF

ELSE // *i* <= 0

IF *j* < 0 THEN

result = (180 + DEGREES(ABS(ATAN(*i* / *j*))))

ELSE // *j*>= 0

result = (360 - DEGREES(ABS(ATAN(*i* / *j*)))) - 180

END IF

END IF

END IF

RETURN result

Here, *i*, *j*, and *k* are the components of the normal vector *n*, determined by three non-collinear points. The calculation method for the normal vector can be implemented in Excel according to the method described in chapter 4.1(1).

## 5 Results and discussions

A variety of fish fossils are found in the middle and upper parts of the Huixingshao Formation of the Llandovery Series, Silurian, in this study area (Fig 2) [17,18]. Researchers have conducted regional geological surveys and section measurements in this study area to acquire basic geological characteristics such as stratigraphic features, lithological features, structural features, and strata thickness. However, it’s difficult to measure attitude, joint attitude, and true thickness of strata in steep sections. Therefore, high-precision 3D real-scene models are applied to measure the attitude and true thickness in these steep sections in the study area, aiming to comprehensively and accurately capture the basic characteristics of the strata bearing fish fossils.

### 5.1 Results of joint attitude measurement

The study area is located at the east wing of Chuanhegai syncline in Youxiu fold belt. stratigraphic joints are highly developed due to the stress from later tectonic activities [38]. We used the high-precision 3D real-scene model to measure the attitudes of 80 stratigraphic joints at target locations in this study (Fig 2, S1 Table). Simultaneously, we used the compass to measure the attitudes of 20 stratigraphic joints near the target location during the field geological survey (Fig 2, S2 Table). This indicates that using high-precision 3D real-scene models for measuring stratigraphic attitude can yield more data that is difficult to measure in the field, and the process of data acquisition is more convenient and efficient.

**Fig 5:**
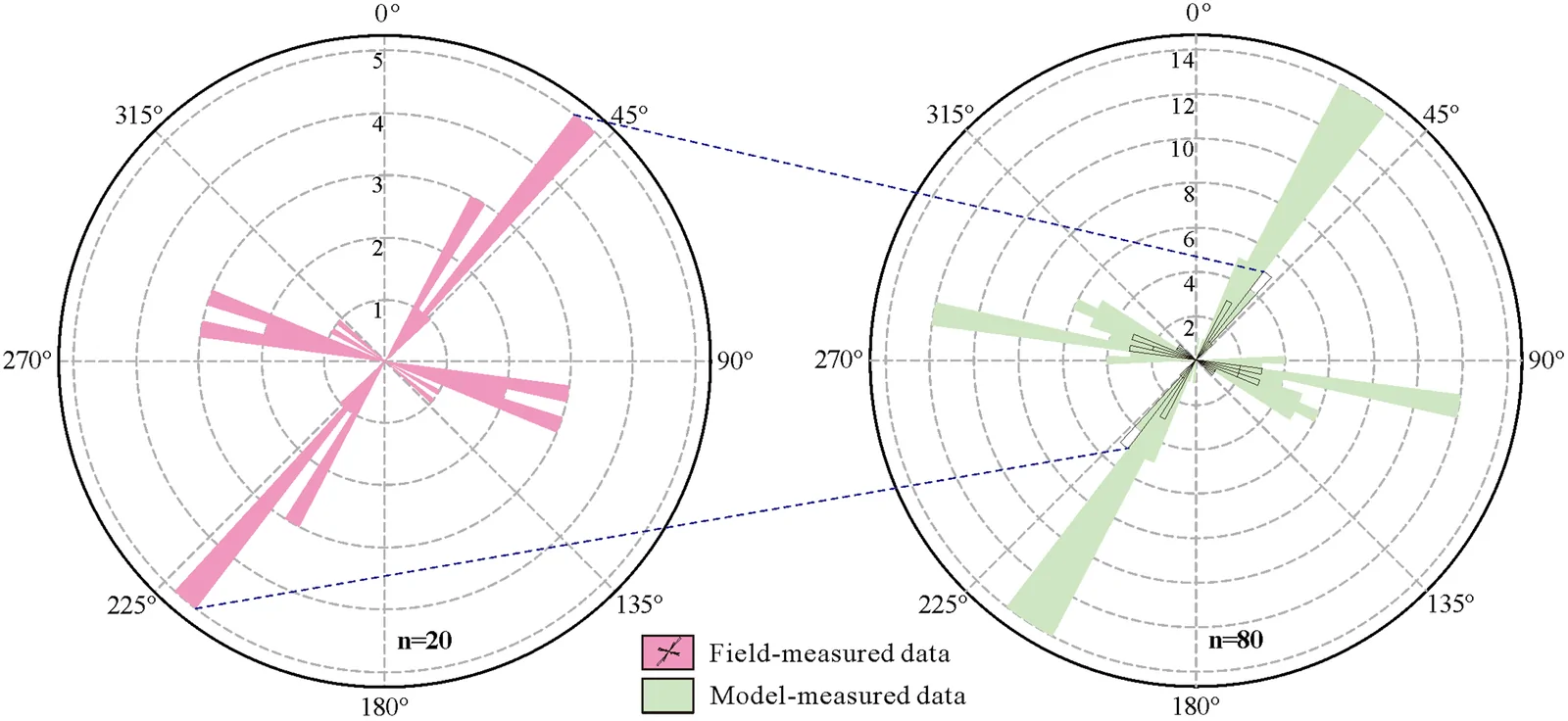
The rose diagrams of joint trend at the target locations in this study. Left figure: The attitudes of 20 stratigraphic joints measured using a compass. Right figure: The attitudes of 80 stratigraphic joints measured using a high-precision 3D real-scene models. The statistical interval of all attitudes is 5 degrees.

According to the measurement results (S1 Table, S2 Table), the stratigraphic joint attitude measurements obtained using high-precision 3D real-scene models differ only slightly from those obtained using a compass. Using high-precision 3D real-scene models, the maximum dip angle measured is 90°, the minimum is 64°, the average is 83°, and the standard deviation is 4.72. Using a compass, the maximum dip angle measured is 88°, the minimum is 77°, the average is 83°, and the standard deviation is 3.37 (Table 1). The average error in dip angle measurements is 0°. The standard deviation of the dip angle measurements obtained using the high-precision 3D real-scene model is larger than that obtained using the compass. This could be attributed to the fact that compass measurements are more susceptible to human factors, potentially leading to deviations from the actual geological conditions and thus exhibiting weaker representativeness compared to the more objective measurements from the 3D model. The data measured using the two methods were plotted on rose diagrams of joint trend (Fig 5). The diagrams show that the results obtained by both methods are largely consistent. However, due to the limited data obtainable with compass measurements, the resulting rose diagrams of joint trends are more scattered, with less distinct dominant orientations. In contrast, the 3D model measurements, providing a larger dataset, yield rose diagrams with clearer dominant orientations, which is more accurate for structural interpretation. Comprehensive analysis indicates that the stratigraphic joint attitude measurements obtained using high-precision 3D real-scene models are largely consistent with those obtained using a compass. Using high-precision 3D real-scene models for measuring stratigraphic joint attitudes offers significant advantages in terms of accuracy and efficiency.

**Table 1:** Statistical table of stratigraphic joint dip angle measurement results in the study area.

Statistical value	3D model measurement	Compass measurement
	*θ*	*θ*
Minimum	64	77
Median	84	84
Maximum	90	88
Average	83	83
Standard deviation	4.72	3.37

Note: *θ* is Dip angle.

### 5.2 Results of strata attitude and true thickness measurement

**Fig 6:**
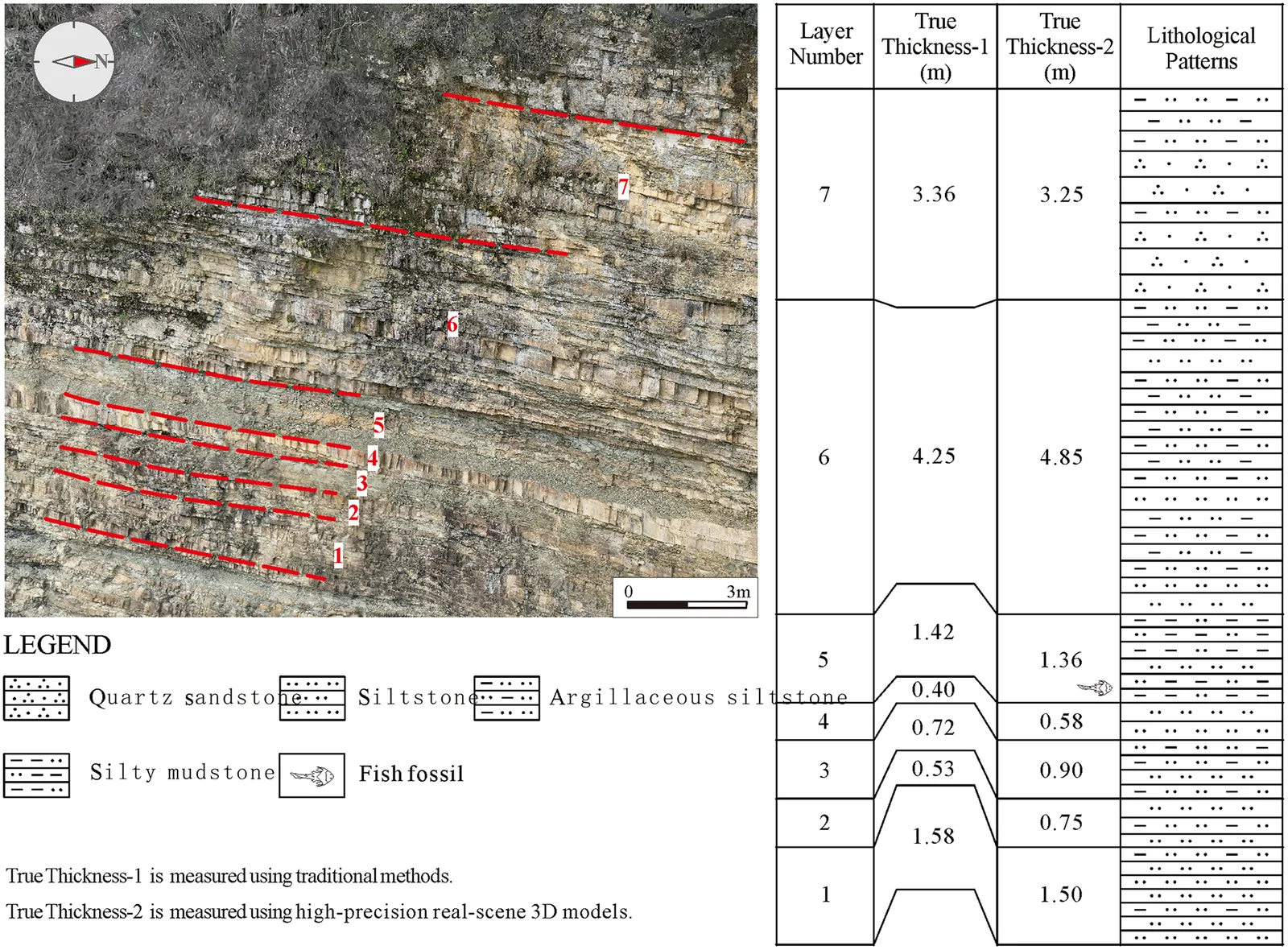
Stratigraphic column chart at the target locations in this study (Scale is 1:100).

It is an important task to measure stratigraphic attitude and true thickness during field geological surveys. Using traditional methods to measure stratigraphic attitude and true thickness is subject to certain errors due to instrument precision, human reading deviations, and field geological conditions. We used high-precision 3D real-scene models to measure the attitudes and true thickness of 7 strata at target locations in this study (S3 Table). We can draw a stratigraphic column chart at a scale of 1:100 based on the measurement results (Fig 6). The error in single-layer true thickness measurement results is 0.06−0.22m compared with the stratigraphic column chart drawn by traditional field measurements. The stratigraphic column chart drawn based on the measurement results from 3D real-scene models can clearly and accurately represent the true thickness and relationships of different strata, aiding in the analysis of the basic geological characteristics of fish fossil-bearing layers.

## 6 Conclusion

The measurement results of strata attitude and true thickness using high-precision 3D real-scene models are reliable compared to traditional methods. The use of 3D real-scene models significantly enhanced the efficiency of geological measurements and reduced fieldwork time. With remote sensing and automated data collection, the research team was able to quickly gather and process extensive geological data, thus shortening the project duration and reducing manpower and material costs.

The high-precision 3D real-scene modeling technology is particularly suitable for use in steep terrains that are difficult to measure directly. This technology overcomes the limitations of traditional geological measurement methods in such terrains, ensuring accurate measurements even under the most challenging topographical conditions. However, it’s important to acknowledge the methodological limitations of this approach. For instance, the resolution of the 3D model can be constrained in very steep terrains, potentially affecting the accuracy of detailed measurements. Additionally, variations in lighting conditions during data acquisition can impact the accuracy of the photogrammetric processing.

Overall, this research validated the feasibility of using high-precision 3D real-scene models in geological measurements. Furthermore, it is important to consider potential error sources in complex environments, such as vegetation-covered areas that can obscure the underlying geological structures and affect the accuracy of UAV photogrammetry. In the future, this technology is expected to be applied in a wider range of geological surveys and resource exploration projects [39,40], especially in scenarios that require rapid and precise measurement of complex geological structures. Future research could also explore the integration of this technology with LiDAR (Light Detection and Ranging) to achieve enhanced precision in measurements and potentially develop automated algorithms for stratigraphic attitude extraction.

## Supporting information

S1 Table The attitudes of joints measured using the high-precision 3D real-scene models at the target locations in the study area. (XLSX)

S2 Table The attitudes of joints measured using the compass at the target locations in the study area. (XLSX)

S3 Table The attitudes and true thicknesses of strata measured using the high-precision 3D real-scene models. (XLSX)
